# Improvement of a MALDI-TOF database for the reliable identification of *Candidozyma auris* (formally *Candida auris*) and related species

**DOI:** 10.1128/spectrum.01444-24

**Published:** 2024-11-19

**Authors:** Hui-Hui Zhu, Miao-Miao Liu, Teun Boekhout, Qi-Ming Wang

**Affiliations:** 1 School of Life Sciences, Institute of Life Sciences and Green Development, Hebei University, Baoding, Hebei, China; 2 College of Sciences, King Saud University, Riyadh, Saudi Arabia; 3 The Yeasts Foundation, Amsterdam, the Netherlands; 4 Hebei Basic Science Center for Biotic Interaction, Hebei University, Baoding, Hebei, China; 5 Engineering Research Center of Ecological Safety and Conservation in Beijing-Tianjin-Hebei (Xiong’an New Area) of MOE, Baoding, Hebei, China; Laboratory Corporation of America Holdings, Burlington, North Carolina, USA

**Keywords:** *Candidozyma auris*, *Candida auris*, *Candidozyma haemuli*species complex, MALDI-TOF, mass spectrometry, yeast, infection, identification

## Abstract

Matrix-assisted laser desorption/ionization time-of-flight mass spectrometry (MALDI-TOF MS) is a promising technique for the rapid identification microorganisms. The aim of this study was to create a new database for the accurate identification of *Candidozyma auris* (formerly known as *Candida auris*) and 11 species of the *Candidozyma haemuli* species complex, including *C. chanthaburiensis*, *C. duobushaemuli*, *C. haemuli*, *C. heveicola*, *C. khanbhai*, *C. konsanensis*, *C. metrosideri*, *C. ohialehuae*, *C. pseudohaemuli*, *C. ruelliae*, and *C. vulturna*. Seventy-one *Candidozyma* isolates from different national institutions were studied. Thirty-seven strains were used to create a MALDI-TOF (microTyper MS) database using the formic acid extraction method. The validation of this database was performed with 34 other strains of the genus *Candidozyma*, and the result was compared with the identification results when using DBRs v1.0.0.4 (Tianrui, China). Our library allowed a 100% identification of the evaluated strains with all strains showing log scores of >2.0. Repeatability and reproducibility tests result showed a coefficient of variation of the log score values of less than 5%. The MALDI-TOF MS system can identify *C. auris* and related species quickly and accurately. This method will play a crucial role in accurately diagnosing infectious agents of the genus *Candidozyma* in clinical practice.

**IMPORTANCE:**

Importance *Candidozyma auris*, also known as *Candida auris*, has quickly spread across the world, and prompt identification of *C. auris* from infected individuals is critical. However, a standard identification method is lacking for the identification of *C. auris* in clinical and public health laboratories. To make matters worse, its biochemical assimilation profile was found to be similar to that of closely related and even no-related species, leading to frequent misidentification. To improve diagnostics of this and closely related species, we created a database of reference mass spectra resulting in the efficient and correct identification of all *Candidozyma* species by matrix-assisted laser desorption/ionization time-of-flight mass spectrometry (MALDI-TOF MS). Moreover, potential pathogenic species of *Candidozyma* can be effectively identified by MALDI-TOF MS, and differentiated from non-clinically relevant phylogenetic relatives. Thus, MALDI-TOF MS may help expedite laboratory diagnosis and treatment of *C. auris* and related species of clinical importance and help the clinician to decide on early treatment.

## INTRODUCTION

Since it was first isolated and described in 2009, *Candidozyma auris*, also known as *Candida auris*, has emerged as a new fungal pathogen causing deep infections and fungemia in at-risk populations, posing a global threat to public health (1, 2). The species is multidrug resistant, highly infectious, and difficult to clear, which are challenges faced in treatment of infections. In 2022, the species was included in the “critical priority” list of fungal pathogens by the World Health Organization. Recently, Liu et al. (3) reassigned *Candida auris* and related species into the newly created genus *Candidozyma* based on a genome-scale approach. *C. auris* is phylogenetically closely related to the *Candidozyma haemuli* (formerly known as *Candida haemulonii*) species complex that includes *C. haemuli* and *C. duobushaemuli*, species that also cause infection (4). Additionally, it was considered a phylogenetic relative of *C. pseudohaemuli* (5). Recently, De Jong et al. (6) described a new species related to the *C. haemuli* species complex, namely *C. khanbhai*. Based on molecular phylogenetic analyses, 12 species are currently placed in the genus *Candidozyma* (3, 6), namely *C. auris*, *C. chanthaburiensis* (7), *C. duobushaemuli*, *C. haemuli*, *C. heveicola* (8), *C. khanbhai*, *C. konsanensis* (9), *C. metrosideri* (10), *C. ohialehuae* (10), *C. pseudohaemuli*, *C. ruelliae* (11), and *C. vulturna* (12). Importantly, several *Candidozyma* species can cause invasive human diseases (13–15).

The increasing number of yeast *Candida* species causing human infections is a challenge for clinical laboratories to provide rapid and reliable identification, especially for closely related *Candida* species (16). The emerging multidrug-resistant yeasts of the genus *Candidozyma* are a significant concern for global public health as they cause major outbreaks in healthcare facilities worldwide (17–20). *C. auris* infection cases have been found in at least 50 countries across 6 continents (18, 21, 22). Additionally, the prevalence rates of species of the *C. haemuli* complex isolates are increasing in the world (23). Some of these *Candidozyma* species have been isolated from natural sources, e.g., *C. vulturna* that was isolated from flowers (12), *C. haemuli* from fish (24), and *C. duobushaemuli* from insects (4). These species were initially isolated and described from natural environments but have now been reported from clinical settings (13, 15, 20). They have been documented to cause vulvovaginal candidiasis (25), invasive (26), and bloodstream infections (27, 28). *C. auris* was first discovered and described from the external ear discharge of a patient in Japan (1). Casadevall et al. (29) hypothesized that before its emergence as a human pathogen, *C. auris* existed as an environmental fungus. In his view, *C. auris* likely adapted to higher temperatures due to global warming and consequently evolved into a human pathogen. Other fungal species, such as *Emergomyces* (formerly placed in the genus *Emmonsia*) (30) and *Cryptococcus neoformans* and *Cryptococcus deuterogattii* (31), can grow at 37°C or higher temperatures (32, 33). It has been suggested that naturally thermotolerant species may have the potential to evolve into human and animal pathogenic species, and studies reported that non-thermotolerant species could become clinically relevant by becoming thermotolerant (34). For example, the acquisition of thermotolerance presumably increases the potential pathogenic of *Metarhizium anisopliae* (35), *Saccharomyces cerevisiae* (36), and *Fusarium* species (37). Therefore, it is worth to be aware of those fungal species that are phylogenetically close relatives of known pathogens but that lack thermotolerance, as these might be considered candidates for the potential emergence of new fungal opportunists. With this background, it is important to include non-clinically relevant phylogenetic relatives, i.e., *C. chanthaburiensis*, *C. heveicola*, *C. konsanensis*, *C. metrosideri*, and *C. ohialehuae*, together with the clinically relevant species of the emerging yeast genus *Candidozyma* in identification protocols. In addition, such non-opportunistic species are a good reference for the clinically relevant ones when setting up any identification system.

To promptly detect and treat patients, it is crucial to identify the infectious strains accurately and promptly, which is a key issue in epidemiological control, hospital hygiene, and the start of early treatment (38). During the last decades, advances in diagnostics and molecular tools have enabled better detection and identification of emerging fungi that are clinically relevant. However, there remain numerous challenges in accurately identifying strains of *C. auris* and related species of the *C. haemuli* species complex. Based on automated commercial identification systems (i.e., VITEK 2, MicroScan, and API 20C AUX), *C. auris* can be misidentified as *C. haemuli*, *Candida famata*, or even the basidiomycetous yeast *Rhodotorula glutinis* (39–41). The prevalence of *C. auris* and related species is probably underestimated due to such unreliable identifications (42). Recently, DNA sequencing and matrix-assisted laser desorption ionization-time of flight mass spectrometry (MALDI-TOF MS) have been considered the most efficient methodologies for the reliable identification of *C. auris* and related species in routine microbiology laboratories (43–45). The use of molecular biological techniques is, however, time-consuming and expensive, and MALDI-TOF MS has become the best choice for routine diagnosis of bacterial and fungal microbial pathogens (46). MALDI-TOF MS compares species-specific protein fingerprints of an infectious agent with similar entries present in a database of reference spectra using various algorithms. With this method, many microorganisms, including yeast, can be rapidly identified (47, 48). Within less than 10 years, the introduction of MALDI-TOF MS has enabled clinical laboratories to quickly and accurately identify microorganisms, including the most clinically relevant fungi (49, 50).

This study aims to improve and evaluate a MALDI-TOF database for identifying *C. auris* and related species of the *C. haemuli* complex that will help to gain a better understanding of their epidemiology and clinical incidence and, above all, will greatly contribute to improved patient care.

## MATERIALS AND METHODS

### Yeast isolates

Seventy-one *Candidozyma* strains used in this study came from different national institutions that were identified in our laboratory. The strains came from clinical and natural environments (Tables 1 and 2). All strains were first cultured on yeast extract peptone dextrose (YPD) medium (10) at 25°C for 48–72 h. Species identification was carried out by amplification and sequencing of the internal transcribed spacer region of the ribosomal DNA as described previously, followed by GenBank basic local alignment search tool (BLAST) pairwise sequence alignment (http://www.ncbi.nlm.nih.gov/BLAST/Blast.cgi) (51). Of 71 isolates, a total of 37 sequence proven isolates (*C. auris*, *n* = 5; *C. chanthaburiensis*, *n* = 1; *C. duobushaemuli*, *n* = 3; *C. haemuli*, *n* = 12; *C. heveicola*, *n* = 1; *C. khanbhai*, *n* = 2; *C. konsanensis*, *n* = 1; *C. metrosideri*, *n* = 1; *C. ohialehuae*, *n* = 1; *C. pseudohaemuli*, *n* = 4; *C. ruelliae*, *n* = 4; *C. vulturna*, *n* = 2) were used for MALDI-TOF MS database creation (Table 1). Thirty-four other strains were used to evaluate the new *C. auris* and related species database (Table 2). To properly evaluate this new database, three other species (*Kodamaea ohmeri* [also known as *Pichia ohmeri*], *n* = 3; *Pichia kudriavzeveii* [also known as *Candida krusei*], *n* = 5; *Meyerozyma guilliermondii* [also known as *Candida guilliermondii*], *n* = 1) were added for database creation, and 53 isolates of 10 other fungal species were included to test the developed database.

**TABLE 1:** Spectra of *Candidozyma* strains used for hierarchical cluster analysis (*n* = 37)

Species	Isolate	Source	Country
*C. haemuli*	G7	Clinic	Beijing, China
*C. haemuli*	C001	Clinic	Shanghai, China
*C. haemuli*	140C-5	Mudflat	China
*C. haemuli*	AFheads13-2	Scalp	Beijing, China
*C. haemuli*	SLLAear3-7	External ear	Beijing, China
*C. haemuli*	SLLAheads7-1	Scalp	Beijing, China
*C. haemuli*	SLLAear3-2	External ear	Beijing, China
*C. haemuli*	AFheads13-1	Scalp	Beijing, China
*C. haemuli*	SLLAheads12-2	Scalp	Beijing, China
*C. haemuli*	AFhead11	Scalp	Beijing, China
*C. haemuli*	JCM3762	Fish	Miami, Florida
*C. haemuli*	SLLAear3-1	External ear	Beijing, China
*C. auris*	SJ01	Urine	Shenyang, China
*C. auris*	2,854	Clinic	Shanghai, China
*C. auris*	2,855	Clinic	Shanghai, China
*C. auris*	S4N83-96	Soil	Sichuan, China
*C. auris*	CS323-6	Soil	Hainan, China
*C. pseudohaemuli*	JCM12453	Blood	Thailand
*C. pseudohaemuli*	2.3703	Insect	Beijing, China
*C. pseudohaemuli*	2.3645	Insect	Beijing, China
*C. pseudohaemuli*	C018	Clinic	Shanghai, China
*C. ruelliae*	2S16-1P2	Soil	South Pole
*C. ruelliae*	HHDS32-1	Seawater	Hainan, China
*C. ruelliae*	SXY51	Rotten wood	Shaanxi, China
*C. ruelliae*	CBS10815	Flower	India
*C. vulturna*	CBS14366	Flower	Philippines
*C. vulturna*	CVDH06	Blood and PICC^*a*^ tip	Shanxi, China
*C. konsanensis*	CBS12666	Flower	Thailand
*C. chanthaburiensis*	CBS10926	Bark	Thailand
*C. heveicola*	CBS10701	Myron	Yunnan, China
*C. metrosideri*	CBS16091	Flower	Hawaii
*C. ohialehuae*	CBS16092	Flower	Hawaii
*C. khanbhai*	CBS16555	Blood	Malaysia
*C. khanbhai*	AFear10	External ear	Beijing, China
*C. duobushaemuli*	C012	Clinic	Shaanxi, China
*C. duobushaemuli*	C013	Clinic	Shaanxi, China
*C. duobushaemuli*	C014	Clinic	Shaanxi, China

^*a*^ PICC, peripherally inserted central catheters.

**TABLE 2:** Thirty-four *Candidozyma* strains were used in this study for mass spectrum profile verification, ordered by species

Species	Isolate	Source	Country
*C. haemuli*	H1	Clinic	Beijing, China
*C. haemuli*	AFheads13-3	Scalp	Beijing, China
*C. haemuli*	SLLAhead4-1	Scalp	Beijing, China
*C. haemuli*	SLLAear3-4	External ear	Beijing, China
*C. haemuli*	SLLAear3-6	External ear	Beijing, China
*C. haemuli*	SLLAheads7-4	Scalp	Beijing, China
*C. haemuli*	42–10-9	Mudflat	China
*C. haemuli*	42–10-8	Mudflat	China
*C. haemuli*	SLLAheads12-1	Scalp	Beijing, China
*C. haemuli*	SLLAheads7-3	Scalp	Beijing, China
*C. haemuli*	SLLAhead1	Scalp	Beijing, China
*C. haemuli*	SLLAear3-8	External ear	Beijing, China
*C. haemuli*	AFheads5-3	Scalp	Beijing, China
*C. haemuli*	AFheads13-4	Scalp	Beijing, China
*C. haemuli*	SLLAear3-3	External ear	Beijing, China
*C. haemuli*	SLLAheads7-2	Scalp	Beijing, China
*C. auris*	CBS12766	Blood	India
*C. auris*	BJCA001	Bronchoalveolar lavage fluid	Beijing, China
*C. auris*	2853	Clinic	Shanghai, China
*C. auris*	2856	Clinic	Shanghai, China
*C. auris*	CBS10913	External ear	Japanese
*C. auris*	CS323-7	Soil	Hainan, China
*C. pseudohaemuli*	2.3280	Bark	Beijing, China
*C. pseudohaemuli*	2.3692	Insect	Beijing, China
*C. pseudohaemuli*	2.3706	Insect	Beijing, China
*C. pseudohaemuli*	2.3241	Insect	Beijing, China
*C. ruelliae*	21S16-8-2P2A	Soil	South Pole
*C. ruelliae*	NJ2S16-72Y	Soil	South Pole
*C. ruelliae*	NJWLNJF8-2Y	Soil	South Pole
*C. ruelliae*	2S16-1-2D	Soil	South Pole
*C. vulturna*	CVDH15	Blood	Shanxi, China
*C. vulturna*	CVDH17	PICC tip	Shanxi, China
*C. khanbhai*	CBS16213	Nose swab	Kuwait
*C. khanbhai*	AFear10-2	External ear	Beijing, China

### MALDI-TOF MS analysis

#### Sample extraction protocol

The strains were sub-cultured on YPD plates and incubated at 25°C for 2–7 days. An ethanol/formic acid method was used for protein extraction (52). Briefly, one or two isolated colonies were picked, and the fungal material was placed in a 1.5 mL micro-centrifuge tube. Three hundred milliliter ultrapure water and 900 mL of anhydrous ethanol were added. The suspension was mixed and centrifuged at 12,000 g for 3 min. After removal of the supernatant, the pellet was air dried for 10 min. Twenty microliter of 70% formic acid was added and mixed by vortexing. Next, 20 µL acetonitrile was added and mixed, and the supernatant containing the extracted protein was acquired after centrifugation (12,000 g for 3 min). One microliter protein extract was placed on a target plate and air-dried, and 1 µL α-cyano-4-hydroxycinnamic acid (Zybio Inc., Jiangsu, China) matrix solution was added. The target plate with samples was dried at room temperature and then placed in the instrument (Zybio Inc., Tianrui, China) for analysis.

#### Generation and analysis of new mass spectrum database for C. auris and related species

Mass spectrometry analysis was performed using a MALDI-TOF MS microTyper MS (Zybio Inc., Tianrui, China). Before the identification used for mass spectrum profile (MSP), the identity of the isolates was confirmed by DNA sequencing. MS spectra were obtained in linear mode within a range of 2,000–20,000 Da and an acceleration voltage of 20 kV. *Escherichia coli* ATCC 25922 was used for mass calibration, with an average deviation of molecular weight less than 300 ppm after correction. MS data were analyzed using Ex-Accuspec software v2.0 (Tianrui, China). As per identification criteria by the manufacturer’s instructions, results were according to the log score values, in particular: ≥2.0, species identification; 1.7–1.99, genus identification; and <1.7, unreliable identification (50, 51, 53).

For MSP dendrogram construction, each sample was coated with 11 targets. At least 24 high-quality spectra with stable baseline, abundant protein peaks and an even distribution were selected. MSP was then loaded into Ex-Smartspec software v 2.0 (Tianrui, China) for dendrogram clustering construction with default settings (distance measure: cosine; clustering: complete), and the composite correlation index (CCI) matrix was used to analyze the correlation between spectra of different isolates.

#### MALDI-TOF database evaluation

For each strain used for testing the new database, spectra from four spots were obtained and compared to entries of the new *C. auris* and related species database and the MALDI-TOF MS in-house Reference Library DBRs v1.0.0.4 (Tianrui, China). The species identification standards were analyzed according to section *Generation and analysis of new mass spectrum database for C. auris and related species*. In this study, the repeatability and reproducibility of the new database were analyzed. To test repeatability, six strains of *C. auris*, *C. haemuli*, *C. khanbhai*, *C. pseudohaemuli*, *C. ruelliae*, and *C. vulturna* were taken from 2-, 3-, 4-, and 5-day-old colonies. For each strain, one protein extract was spotted 30 times on the same steel target plate to evaluate the repeatability of the new database. To test the repeatability of the full protocol, three strains of *C. auris*, *C. haemuli*, and *C. khanbhai*, were taken from 2-, 3-, 4-, and 5-day-old colonies. At the same time, from the same culture, 15 extracts were acquired and spotted on the target plate. To test reproducibility with four strains of *C. auris*, *C. haemuli*, *C. pseudohaemuli*, and *C. ruelliae*. For each strain, 10 successive cultures were taken, and single culture extracts from 2-, 3-, 4-, and 5-day-old colonies were analyzed.

### Statistical analysis

The coefficients of variation (CVs) of the repeatability and reproducibility were measured by Kruskal Wallis test analysis using SPSS (IBM SPSS statistics 22) software. A *P* value of <0.05 was considered statistically significant.

## RESULTS

To verify and evaluate the accuracy and sensitivity of the newly established mass spectrometry database, MSPs of 34 *Candidozyma* isolates were obtained. They were compared to both the newly made database and DBRs v1.0.0.4 (Tianrui, China), which contains three *Candidozyma* species (≥5 strains of *C. auris*, 3 strains of *C. haemuli*, and ≥5 strains of C. *duobushaemuli*). The new database gave correct species identification with scores ≥2.0 for all *Candidozyma* isolates/strains (Table 3). The DBRs v1.0.0.4 (Tianrui, China) gave incorrect species with scores <2.0 for *C. auris*, *C. pseudohaemuli*, *C. ruelliae*, *C. vulturna*, and *C. khanbhai*. For the species *C. haemuli*, a correct species ID (score ≥2.0) was achieved for three isolates (19%), while 13 isolates were assigned to that species with a lower score between 1.77 and 1.89. Potential false positives from non-*Candidozyma* strains, such as *Clavispora lusitaniae* (also known as *Candida lusitaniae*), *Candida albicans*, *Diutina catenulata* (also known as *Candida catenulata*), *Kodamaea ohmeri*, *Saccharomyces cerevisiae*, *Candida parapsilosis*, *Candida tropicalis*, *Pichia kudriavzeveii*, *Meyerozyma guilliermondii*, and *Nakaseomyces glabrata* (also known as *Candida glabrata*) were evaluated. No misidentification was observed with the new database when tests were run on other yeasts (Table 3).

**TABLE 3:** Comparison of the results of species identification of *C. auris* and related species isolates by reference library DBRs and the newly generated database (*n* = 87)

Species	Evaluation set	Reference library DBRs			The new database		
	N0. of isolates	Log score (<1.7)	Log score (1.7–1.99)	Log score (≥2.0)	Log score (<1.7)	Log score (1.7–1.99)	Log score (≥2.0)
*C. auris*	6	4	2	0	0	0	6
*C. haemuli*	16	0	13	3	0	0	16
*C. khanbhai*	2	2	0	0	0	0	2
*C. pseudohaemuli*	4	4	0	0	0	0	4
*C. ruelliae*	4	4	0	0	0	0	4
*C. vulturna*	2	2	0	0	0	0	2
*Clavispora lusitaniae*	1	0	0	1	1	0	0
*Candida albicans*	1	0	0	1	1	0	0
*Diutina catenulata*	1	0	0	1	1	0	0
*Kodamaea ohmeri*	6	0	1	5	0	0	6
*Saccharomyces cerevisiae*	7	0	0	7	7	0	0
*Candida parapsilosis*	9	0	0	9	9	0	0
*Candida tropicalis*	11	0	0	11	11	0	0
*Pichia kudriavzeveii*	11	0	0	11	0	0	11
*Meyerozyma guilliermondii*	3	0	0	3	0	0	3
*Nakaseomyces glabrata*	3	0	1	2	3	0	0
Total	87	16	17	54	33	0	54

**Fig 1:**
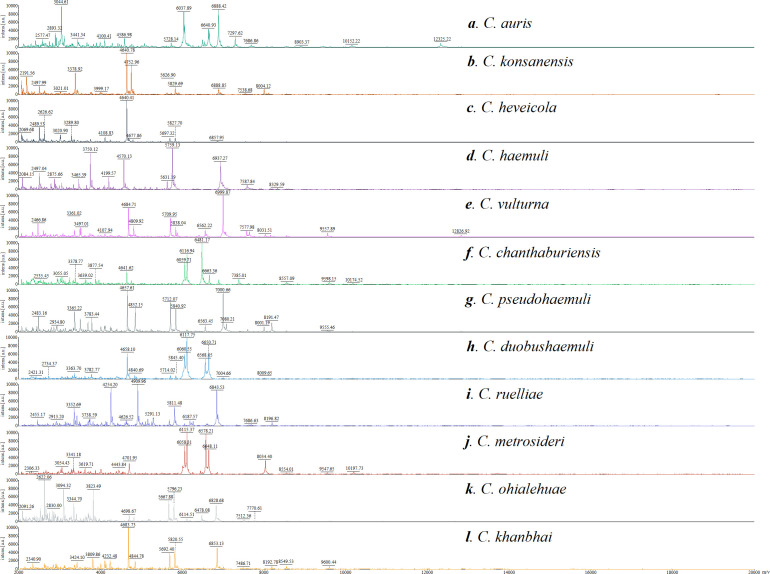
Comparison of spectra obtained for the 12 species of the genus *Candidozyma*, namely *C. auris* (a), *C. konsanensis* (b), *C. heveicola* (c), *C. haemuli* (d), *C. vulturna* (e), *C. chanthaburiensis* (f), *C. pseudohaemuli* (g), *C. duobushaemuli* (h), *C. ruelliae* (i), *C. metrosideri* (j), *C. ohialehuae* (k), and *C. khanbhai* (l).

**Fig 2:**
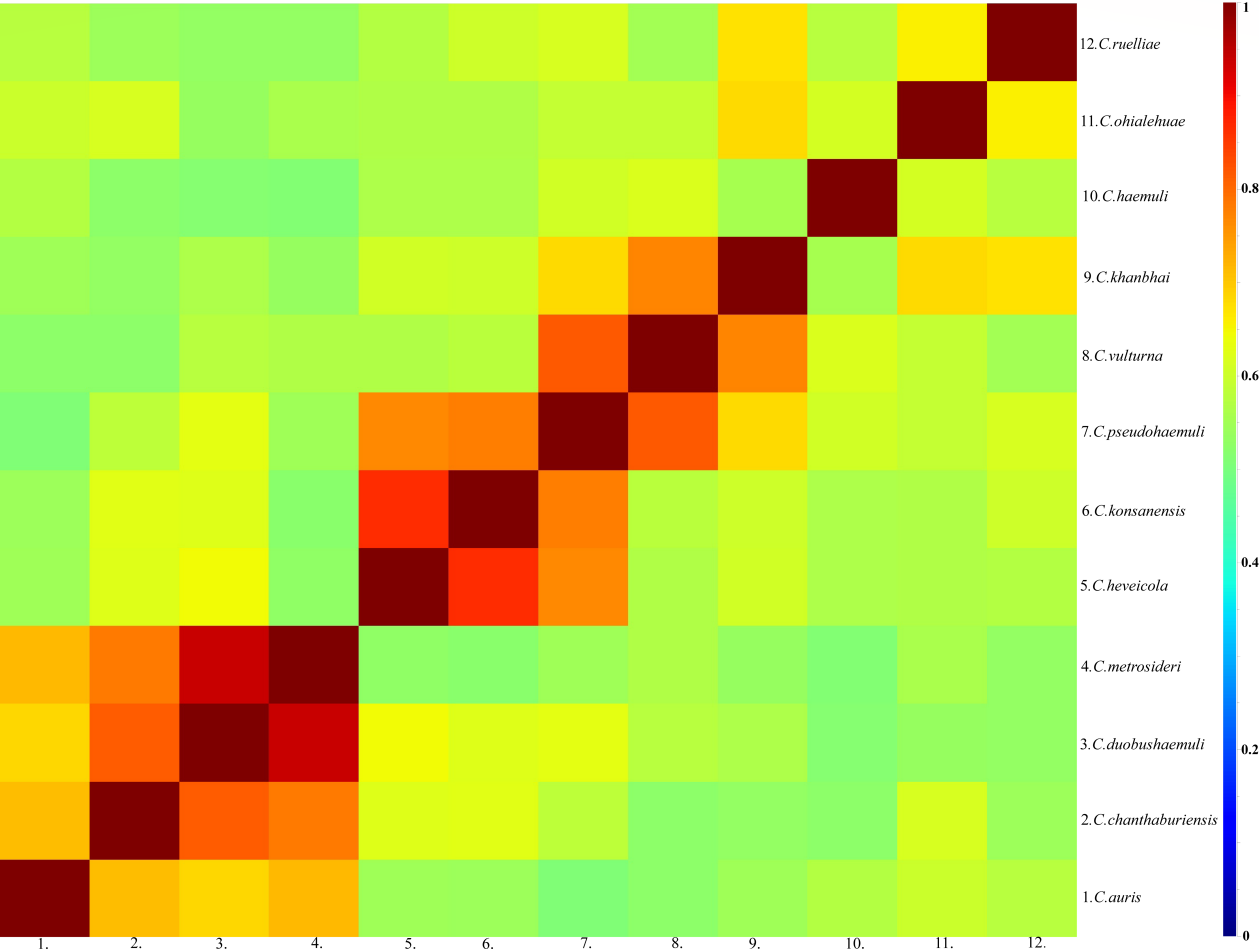
Graphical representation of CCI scores calculated for each species. Blue to green represent CCI scores ranging from 0 to 0.5 (weak similarity). Yellow to red represent CCI scores from 0.5 to 1 (high similarity).

**Fig 3:**
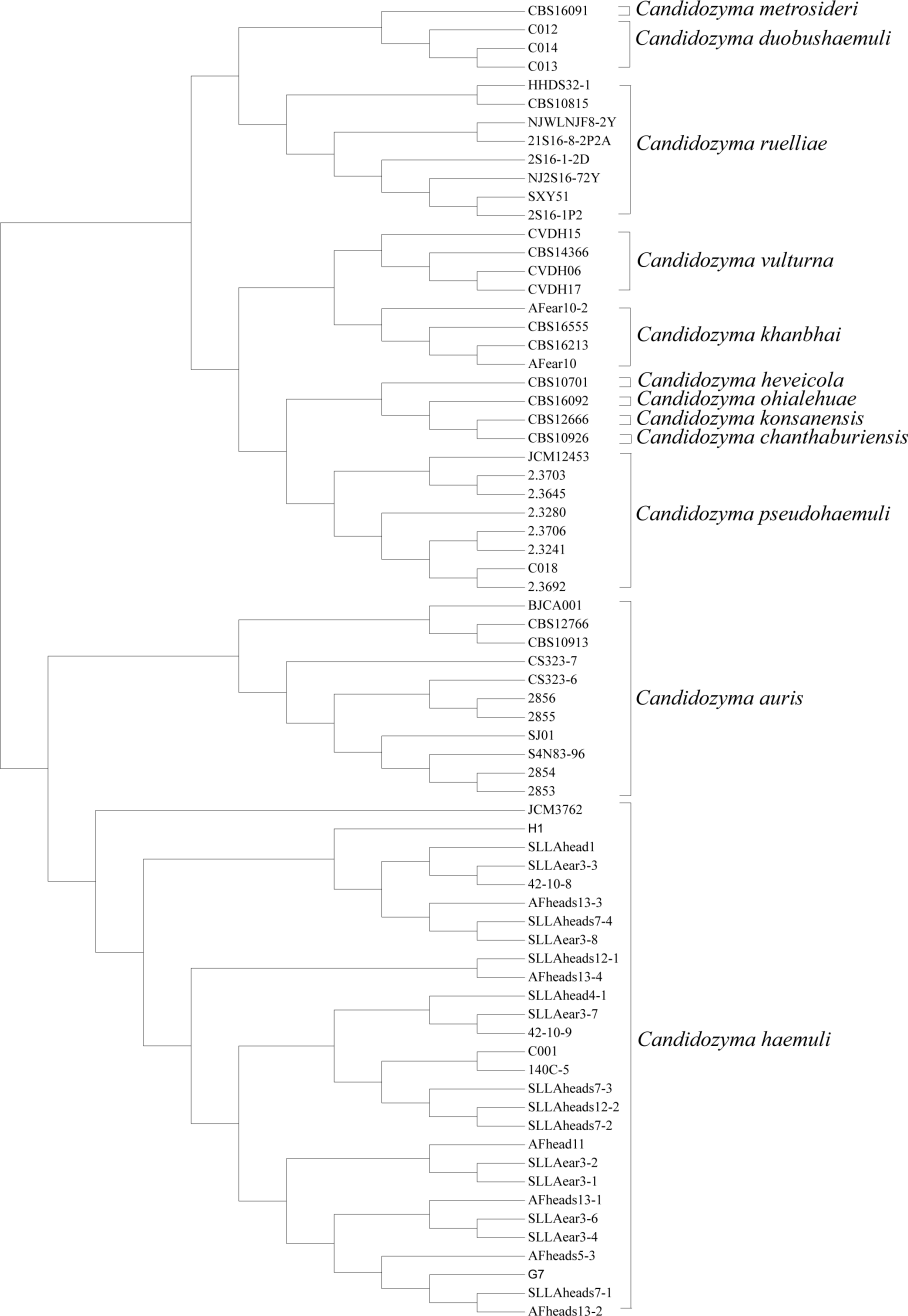
Mass spectrum dendrogram grouping *C. auris*, *C. chanthaburiensis*, *C. duobushaemuli*, *C. haemuli*, *C. heveicola*, *C. khanbhai*, *C. konsanensis*, *C. metrosideri*, *C. ohialehuae*, *C. pseudohaemuli*, *C. ruelliae*, and *C. vulturna* isolates to the species level.

To examine species specificity and compare the spectra of different species, differences in the intensity and the position of the m/z of the spectra of the species were observed (Fig. 1). They exhibited significant differences between the fungal species, and the low similarity between the MSPs of different species is also shown by the CCI analysis of the spectra (Fig. 2). The highest similarity between spectra was observed between *C. duobushaemuli* and *C. metrosideri*, with a CCI of 0.94, which may lead to the misidentification of their in clinical testing. Hierarchical cluster analysis of the MSP confirmed the distribution of the MSPs into species clusters (Fig. 3). The spectra showed sufficient differences between all the species at the species level to allow good identification.

The reproducibility and repeatability and full-protocol tests were analyzed, and the results showed that the CVs calculated based on the identification scores were below 5% (Table 4). For the repeatability and full protocol tests, the average CVs were 1.23% (range, 0.33%–2.22%) and 1.95% (range, 0.78%–2.86%), respectively. For the reproducibility tests, the average CVs was 2% (range, 0.58%–3.09%). Thus, our new database has a high accuracy and stability allowing the reliable identification of *C. auris* and related species of the *C. haemuli* species complex.

**TABLE 4:** Means of the CV of the log score values of the reproducibility and repeatability tests

Age of colony (days)	Mean (range) CV (%) of log score values		
	Analysis repeatability	Full-protocol repeatability	Reproducibility
2	1.16 (0.46–2.16)	1.81 (1.28–2.08)	1.86 (0.58–2.50)
3	1.29 (0.35–2.13)	1.99 (1.71–2.42)	1.97 (1.66–2.48)
4	1.15 (0.33–2.05)	2.08 (0.95–2.77)	2.03 (1.21–2.31)
5	1.31 (0.36–2.22)	1.92 (0.78–2.86)	2.14 (0.65–3.09)

## DISCUSSION

*C. auris*, also known as *Candida auris*, and related species are part of a complex of emerging multidrug-resistant yeast pathogens that recently caused major outbreaks in many countries worldwide (15, 19, 54). Despite the usefulness of MALDI-TOF MS for the identification of various fungi, including yeasts, has been authenticated by many studies (45, 55–58), its use to distinguish between *C. auris* and related species is less well documented. Prakash et al. (59) and Grenfell et al. (42) were able to accurately identify *C. auris* and members of the *C. haemuli* species complex by MALDI-TOF MS (Bruker Daltonics, Bremen, Germany), respectively. Like other studies in which the MALDI Biotyper version 3 database (Bruker Daltonics, Bremen, Germany) successfully differentiated *C. auris*, *C. haemuli*, *C. haemuli* var. *vulnera*, and *C. duobushaemuli* (39), other closely related species of *Candidozyma*, such as *C. pseudohaemuli* and *C. vulturna*, were not included. To accurately identify *C. auris* and related species and reduce diagnostic errors in the clinic, we built a MALDI-TOF database for *C. auris* and all of its phylogenetically related species of the *C. haemuli* species complex. The obtained MSPs were species-specific and had sufficient discrimination to accurately identify *C. auris* and related species at the species level.

The present study reports the evaluation of MALDI-TOF MS for the identification of 12 species of the *C. haemuli* species complex, including the newly identified species *C. khanbhai* (6). Bao et al. (45) created a novel MALDI-TOF database using a Bruker Biotyper (Bruker, Billerica, MA) for the identification of *C. auris*, including 22 strains (five species), and Ceballos-Garzon et al. (60) incorporated 30 strains of *C. auris* in the MALDI-TOF database (Bruker Daltonics, Bremen, Germany). Compared to previous studies, this is the first study that covered *C. auris* and all phylogenetically related species of the genus *Candidozyma*, showing reliable use for clinical diagnosis. This study identified *Candidozyma* at the species level, and the identification scores of all tested strains were above 2.0. Similarly, the study by Bao et al. (45) and Ceballos-Garzon et al. (60) reported identification log scores >2 for all *C. auris* isolates. Also, Grenfell et al. (42) reported identification of all *C. haemuli* isolates with log score >2. We included *C. pseudohaemuli* (*n* = 8) strains for database construction (*n* = 4) and identification (*n* = 4) that resulted in identification log scores >2, compared to the previous study (database construction, *n* = 2, 2) (39, 42). Our identification rate for *C. duobushaemuli* was 100%, compared to the rate of 69% obtained by Grenfell et al. (42). It should be noted that among the 34 *Candidozyma* strains tested in this study, except for *C. auris* and *C. haemuli*, none of them were correctly identified when using DBRs v1.0.0.4 (Tianrui, China), not even at the genus level. This had an obvious reason as the DBRs database does not include spectra of these species. From our research, it became clear that the upgraded database can be successfully applied to the identification of *C. auris* and related species. Therefore, adding internal spectra can better capture the proteomic changes in the MALDI-TOF MS database (58).

Testing the repeatability and reproducibility of the newly established database resulted in CVs value of <5%, indicating that the new database identification results have good stability and accuracy. Similar to our research results, Denis et al. (51) developed a new MALDI-TOF database (Microflex mass spectrometer, Bruker Daltonics, Germany) of *Malassezia* with CVs value of <10%. Moreover, we recognize that our MALDI-TOF database of *C. auris* and related species has limitations, with only one representative included of *C. konsanensis*, *C. chanthaburiensis*, *C. heveicola*, *C. metrosideri*, and *C. ohialehuae*, as these species are not easily available from laboratory collections (7–10). Furthermore, the number of test strains from different geographical locations was relatively small and did not include and analyze the phylogenetic clades of *C. auris*. One study on MALDI-TOF MS spectral profiling showed evidence of geographical clustering for *C. auris* (59). Similarly, Ceballos-Garzon et al. (60) compared the mass spectra of BDAL (Bruker) with the “*C. auris* Colombia library.” They observed a clear separation between the Colombian and the Eastern strains depicted in their dendrogram that showed two clades. Therefore, it is important to analyze whether the various geographical lineages of *C. auris* may impact the accuracy of its identification. Additional strains belonging to these species should be included when available to expand the database in future studies.

The MALDI-TOF MS is a reliable technique that will assist in the rapid and accurate identification of *C. auris* and related species. It has the advantages of fast recognition and high accuracy when compared with other routine methods (61). The low running costs and the rapid, easy-to-operate technology may help to expedite clinical diagnosis and treatment of *C. auris* and related species, although the equipment itself requires a substantial initial investment (58). In addition, developers need to add more MPSs representing the variation present in *Candidozyma* species to expand the database in future research in order to improve its recognition rate.

## Data Availability

The data presented in this study are available on request from the corresponding author.
